# A synthetic targeted RNA demethylation system based on CRISPR‐Cas13b inhibits bladder cancer progression

**DOI:** 10.1002/ctm2.734

**Published:** 2022-02-27

**Authors:** Ganglin Su, Lin Yao, Xiaohong Han, Xinhui Liao, Jieqing Chen, Tao Yin, Ying Dong, Yanfeng Li, Kun Hu, Junwen Xiao, Yuchen Liu, Weiren Huang, Hongbing Mei

**Affiliations:** ^1^ Department of Urology The First Affiliated Hospital of Shenzhen University, Shenzhen Second People's Hospital Shenzhen China; ^2^ Shantou University Medical College Shantou China; ^3^ Key Laboratory of Medical Reprogramming Technology The First Affiliated Hospital of Shenzhen University, Shenzhen Second People's Hospital Shenzhen China; ^4^ Guangdong Key Laboratory of Systems Biology and Synthetic Biology for Urogenital Tumors The First Affiliated Hospital of Shenzhen University, Shenzhen Second People's Hospital Shenzhen China; ^5^ Department of Urology Peking University First Hospital Beijing China

To the Editor:

The N6‐methyladenosine (m6A) modification of messenger RNA (mRNA) is emerging as a key regulator of gene expression, influencing a variety of developmental and biological processes, with m6A homeostasis also being associated with cancer.^1^ Here, we constructed a targeted MYC mRNA demethylation (MYCdm6A) system, and identified MYCdm6A to be capable of inducing a robust demethylation of MYC mRNA and to effectively inhibit the transcription and expression of MYC.

The tumour‐promoting factor MYC plays an important role in the occurrence and development of tumours.^2^ Search results on TCGA show the elevated expression of MYC is negatively correlated with the overall survival (OS) of bladder urothelial carcinoma (BLCA) patients, indicating a poor prognosis for BLCA patients with high MYC expression (Figure S1A). Moreover, inspired by the finding that targeting MYC methylation in cancer cells could serve as a promising and specific strategy for cancer therapy, and the observation of the important role of MYC mRNA methylation in bladder cancer (BCa),^3^, ^4^ we set out to construct a targeted MYC demethylation system, ‘MYCdm6A’. Demethylase fat mass and obesity‐associated protein (FTO) was fused to the C‐terminus of catalytically inactive Cas13b to generate a dCas13b–FTO fusion protein. As m6A readers occur in both the cytoplasm and nucleus, dCas13b–FTO localisation in one of these two regions may have a biological effect different from that in the other region. Therefore, a nuclear export signal was added to the fusion protein to stimulate nuclear export. We incorporated the EF1A promoter to drive gene expression. Following this, we inserted MYCdm6a and the corresponding control cDNA into lentivirus vectors and used the lentiviruses to transport the constructs into cells. Immunofluorescence staining revealed that the fusion protein was detected in both the cytoplasm and nucleus, while endogenous FTO was nuclear localised in human cells (Figure S1B–D).^5^

To assess whether MYCdm6A is capable of inducing MYC mRNA demethylation, we introduced MYCdm6A and a negative control into BCa cell lines 5637 and SW780 and selected the subclones. The results of MeRIP‐PCR supported the relative decrease in the m6A alteration of MYC in BCa cells, indicating that MYCdm6A did induce MYC mRNA demethylation (Figure 1A). Next, we investigated the impact of the demethylation on MYC abundance and mRNA stability. RT‐qPCR and Western blotting showed a downregulation of both the mRNA and protein levels of MYC (Figure 1B,C). Further, we used MYC luciferase reporter assays to confirm that MYCdm6A inhibited MYC translation efficiency (Figure 1D). Changing m6A deposition increases or decreases the expression of methylated mRNAs by controlling mRNA degradation.^6^ To verify whether MYCdm6A induces mRNA degradation, we examined the stability of MYC mRNA and confirmed that MYCdm6A reduced the half‐life of MYC mRNA (Figure 2E). Therefore, we hypothesised that MYCdm6A decreased the binding between MYC mRNA and YTHDF1, an m6A reader protein family member, which promotes MYC stability through an m6A‐dependent mechanism.^7^ By performing RIP‐qPCR, we further revealed that YTHDF1 and MYC mRNA bind to each other in BCa cells, and that this interaction was reduced in tandem with MYCdm6A demethylation (Figure S2).

**FIGURE 1:**
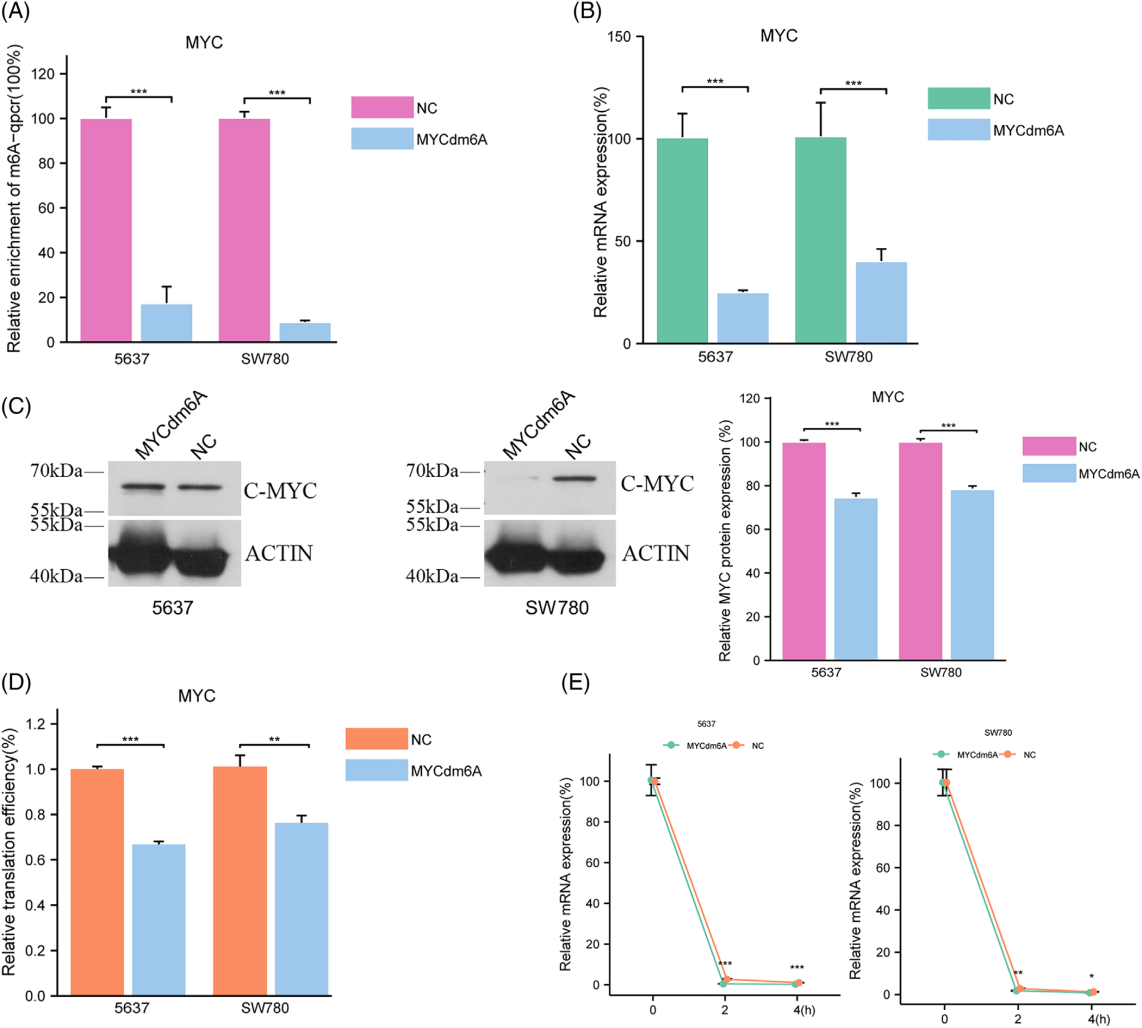
MYCdm6A decreases N6‐methyladenosine (m6A) enrichment (A), abundance (B and C), translation efficiency (D) and stability (E) of MYC in 5637 and SW780 cells. Data are shown as mean ± SEM (*p < .05, **p < .01, ***p < .001, ****p < .001)

**FIGURE 2:**
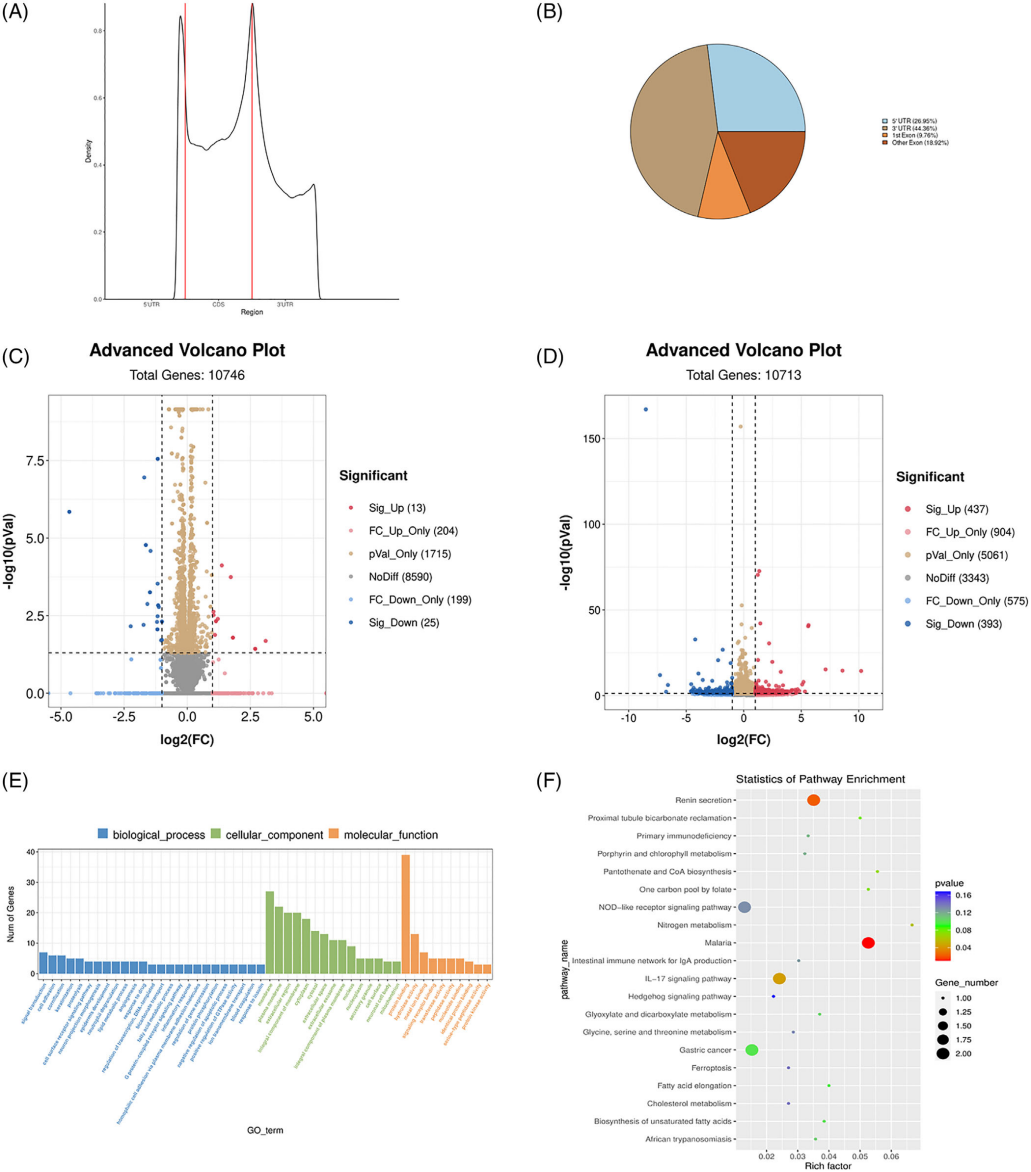
MYCdm6A specificity and nonspecificity in N6‐methyladenosine (m6A) demethylation. (A) M6A peak distribution across the length of messenger RNA (mRNA) transcripts. (B) Distribution of differential m6A peaks diffpeaks across the length of mRNA transcripts. (C) Volcano plot displaying genes with a significant change in the MYCdm6A group compared to the negative control group (|fold change| > 2, *p* < .05); FC, fold change. (D) Volcano plot displaying m6A peaks with a significant change in the MYCdm6A group compared to the negative control group (|fold change| > 2, *p* < .05); FC, fold change. (E) Gene ontology (GO) enrichment study of m6A peaks with a significant change in the MYCdm6A group compared to the negative control group. (F) Kyoto Encyclopedia of Genes and Genomes (KEGG) pathway enrichment analysis of m6A peaks with a significant change in the MYCdm6A group compared to the negative control group

Considering that MYCdm6A is mediated by sgRNA, it was necessary to evaluate whether sgRNA‐guided demethylation had any off‐target effects. To investigate this, we performed m6A sequencing and compared the entire m6A methylomes of MYCdm6A‐transfected and negative control BCa cells. mRNA sequencing was also carried out to assess possible off‐target effects of MYCdm6A on the transcriptome. m6A peaks were concentrated around the stop codons in the 3′‐UTRs of these mRNAs (Figure 2A). Differential m6A peaks around stop codons were enriched in the mRNA 3′‐UTRs (Figure 2B). There were 25 significantly downregulated and 13 significantly upregulated transcripts in the MYCdm6A group compared with the negative control group, among the 10 746 detected transcripts (Figure 2C). Gene ontology (GO) analysis of the MYCdm6A‐induced variegated mRNA revealed that they are connected to protein binding (Figure S3). In addition, there were 393 significantly downregulated and 437 significantly upregulated m6A peaks (*p* < .05) in the MYCdm6A group compared with the negative control group, among the 10 713 detected m6A peaks in the transcriptome (Figure 2D). Moreover, different m6A peaks showing a significant change (*p* < .05) may be related to protein binding and nitrogen metabolism, according to GO and Kyoto Encyclopedia of Genes and Genomes (KEGG) pathway enrichment analyses (Figure 2E,F). The low numbers of affected peaks and genes found in the various clusters showed that the off‐target effects of our tool were minimal.

Utilizing MYCdm6A in BCa cells, we further investigated the effect of suppressed endogenous MYC using a series of functional assays. Flow cytometry and caspase‐3 assay/ELISA were used to assess cell apoptosis, and the targeting of MYC by MYCdm6A was found to substantially enhance cell death in BCa cell lines 5637 and SW780 (Figure 3A,B). Next, a wound‐healing experiment revealed that the MYCdm6A targeting of MYC suppressed the migration of both lines of bladder cancer cells (Figure 3C). Finally, the cell proliferation assay indicated that the MYCdm6A targeting of MYC significantly inhibited the proliferation of BCa cell lines (Figure S4).

**FIGURE 3:**
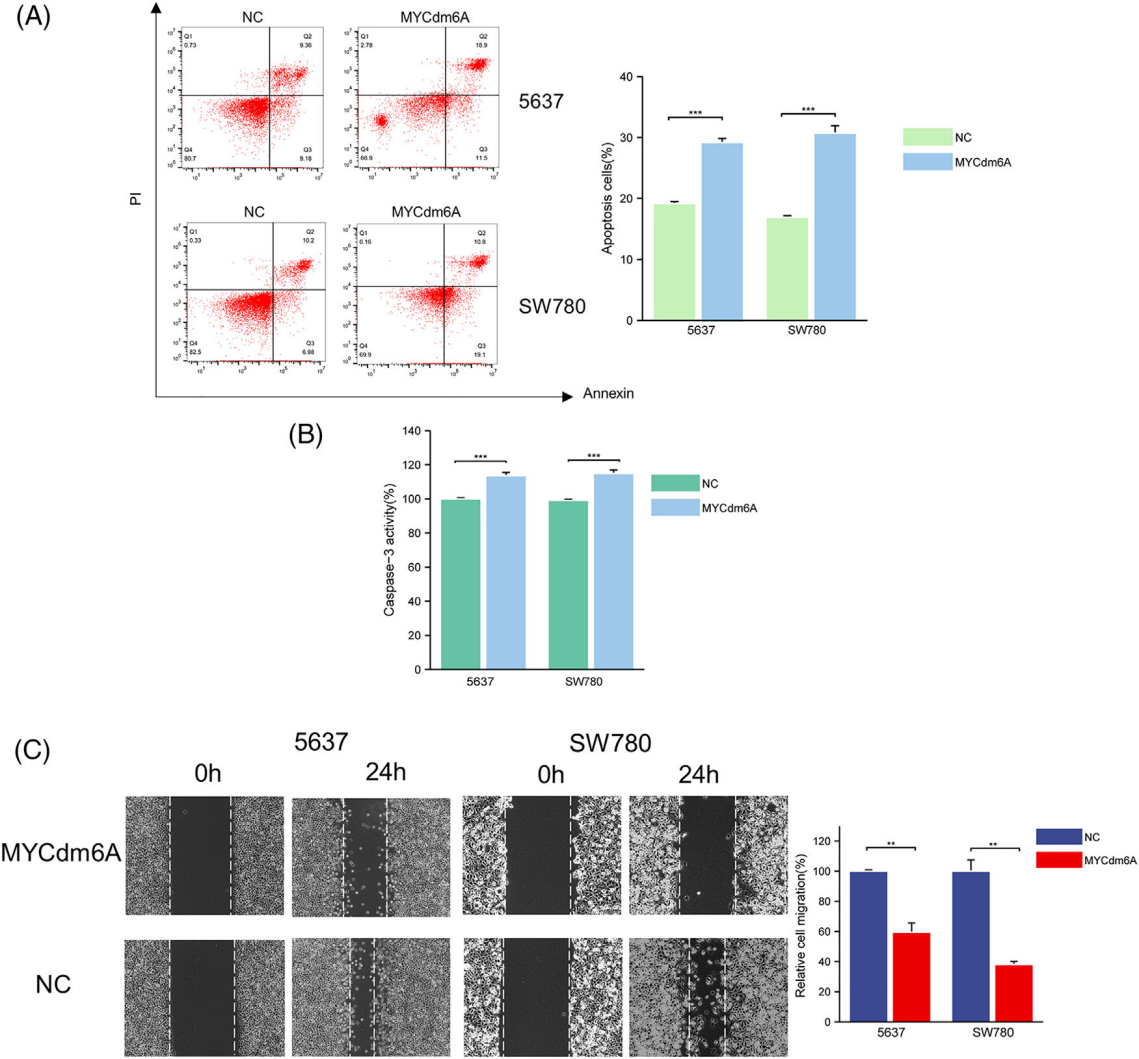
MYCdm6A‐induced apoptosis and inhibited migration of bladder cancer (BCa) cell lines in vitro. (A) Flow cytometry was used to assess the changes in apoptosis of bladder cancer cell lines in vitro. (B) Caspase‐3 ELISA assay was used to assess the changes in apoptosis of bladder cancer cell lines in vitro. (C) Wound healing assays were used to assess the migrations of bladder cancer cell lines in vitro. Data are shown as mean ± SEM (*p < .05, **p < .01, ***p < .001, ****p < .001)

To evaluate the ex vivo therapeutic benefits of MYCdm6A, xenograft models were established by injecting stable SW780 cells expressing MYCdm6A and matching negative control vectors into subcutaneous tissues of nude mice. At the injection location, all of the nude mice developed xenogeneic tumours. Tumour volume was measured twice a week, and tumour mass was weighed on day 29. The weights and volumes of the xenografted tumours were lower in the MYCdm6A group than in the negative control group (Figure 4A–D). Tumours transfected with MYCdm6A‐containing cells showed more widespread necrotic regions than those transfected with negative control cells. Immunohistological examination of MYC and Ki67 in tumour sections showed that both were considerably lower in tumours from the MYCdm6A group than in those from the control group (Figure 4E).

**FIGURE 4:**
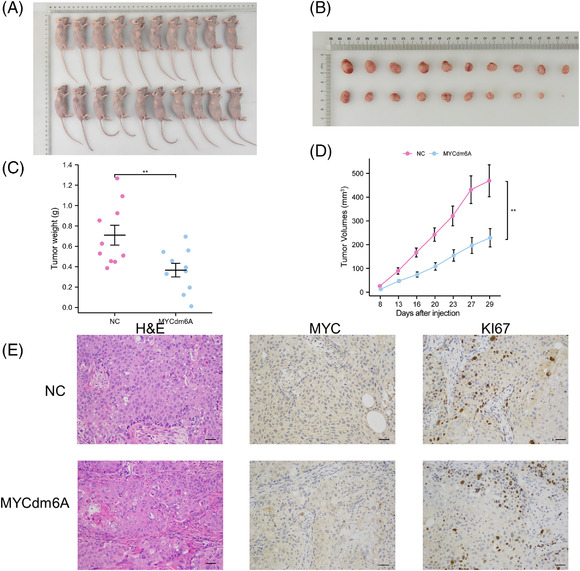
MYCdm6A decreased bladder urothelial carcinoma (BLCA) tumour growth ex vivo. (A) Representative images of xenograft in nude mice. (B) Representative images of xenograft tumours. (C) Tumour weights were determined at day 29. (D) Growth curves of xenograft tumours in the nude mice. (E) Representative pictures for haematoxylin and eosin (H&E) staining and immunohistochemical analysis of MYC and KI67 in the MYCdm6A group and the negative control group. All scale bars: 50 μm. Data are shown as mean ± SEM (*p < .05, **p < .01, ***p < .001, ****p < .001)

In conclusion, our team constructed an antitumor biodevice, MYCdm6A, with the ability to cause a robust demethylation of MYC mRNA, effectively inhibiting MYC transcription and expression. Significantly, both ex vivo and in vitro, this biodevice partially suppressed the malignant phenotypes of BCa cells. MYCdm6A provides a novel strategy for cancer therapy and could be a useful ‘weapon’ against cancer cells due to its transient nature, which eliminates the concern of introducing permanent DNA alterations.

## CONFLICT OF INTEREST

The authors declare that there is no conflict of interest.

## Supporting information

SUPPORTING INFORMATION

SUPPORTING INFORMATION

SUPPORTING INFORMATION

SUPPORTING INFORMATION

SUPPORTING INFORMATION
