# Supplementary figures and images for: A synthetic targeted RNA demethylation system based on CRISPR‐Cas13b inhibits bladder cancer progression
Source: Clin Transl Med. 2022 Feb 27;12(2):e734. doi: 10.1002/ctm2.734 (PMC8882238; doi:10.1002/ctm2.734)

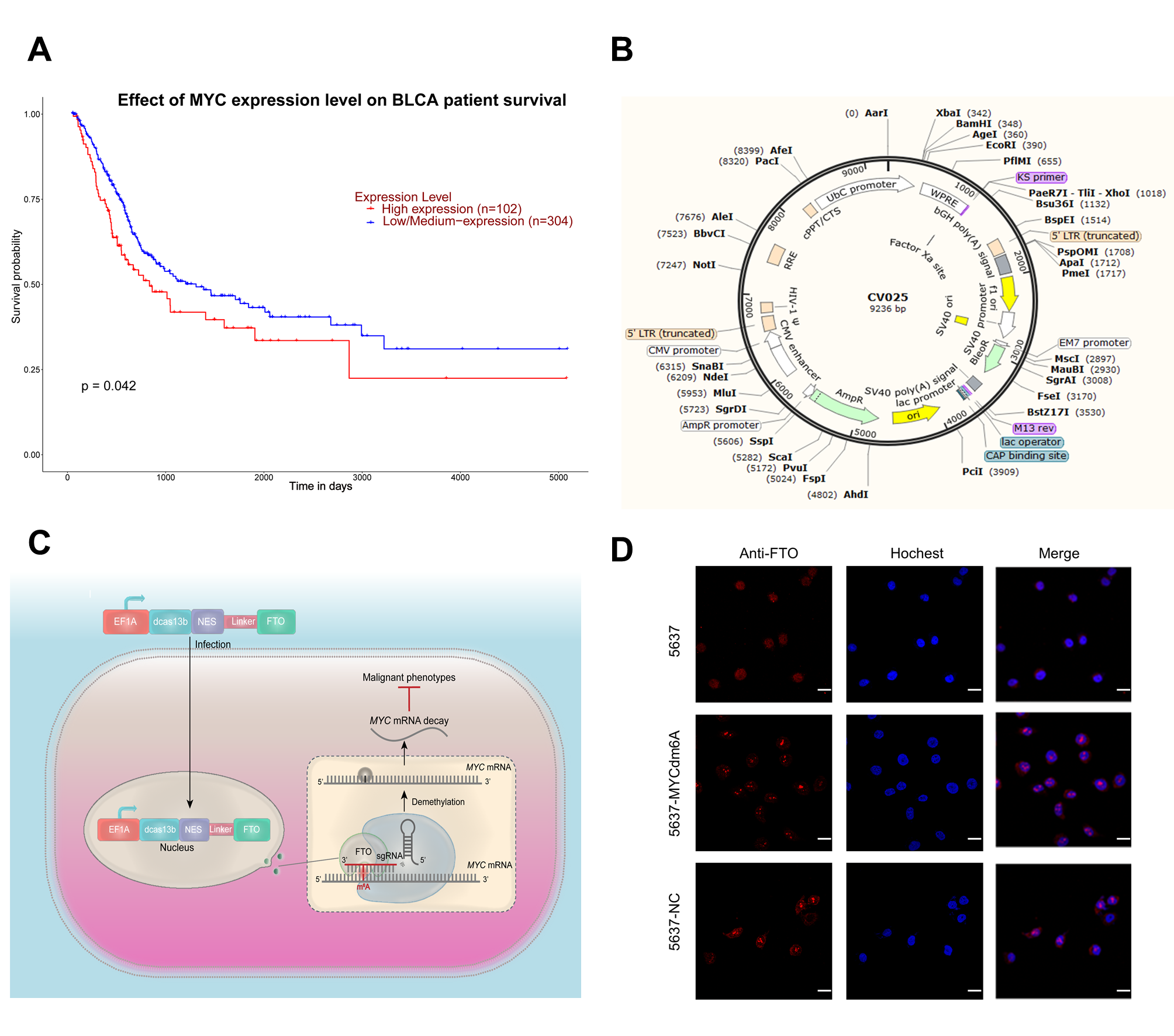

Supplement: Supplementary file 1 — SUPPORTING INFORMATION [file CTM2-12-e734-s003.tif]

# YTHDF1 RIP PCR

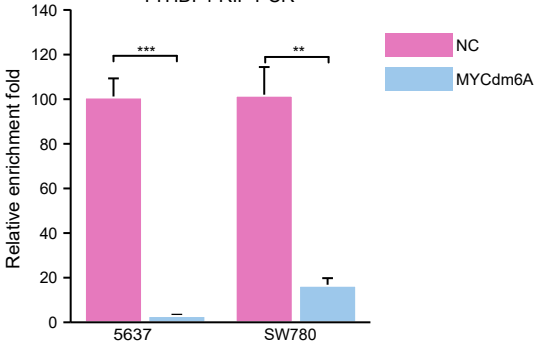

Supplement: Supplementary file 2 — SUPPORTING INFORMATION [file CTM2-12-e734-s005.pdf]

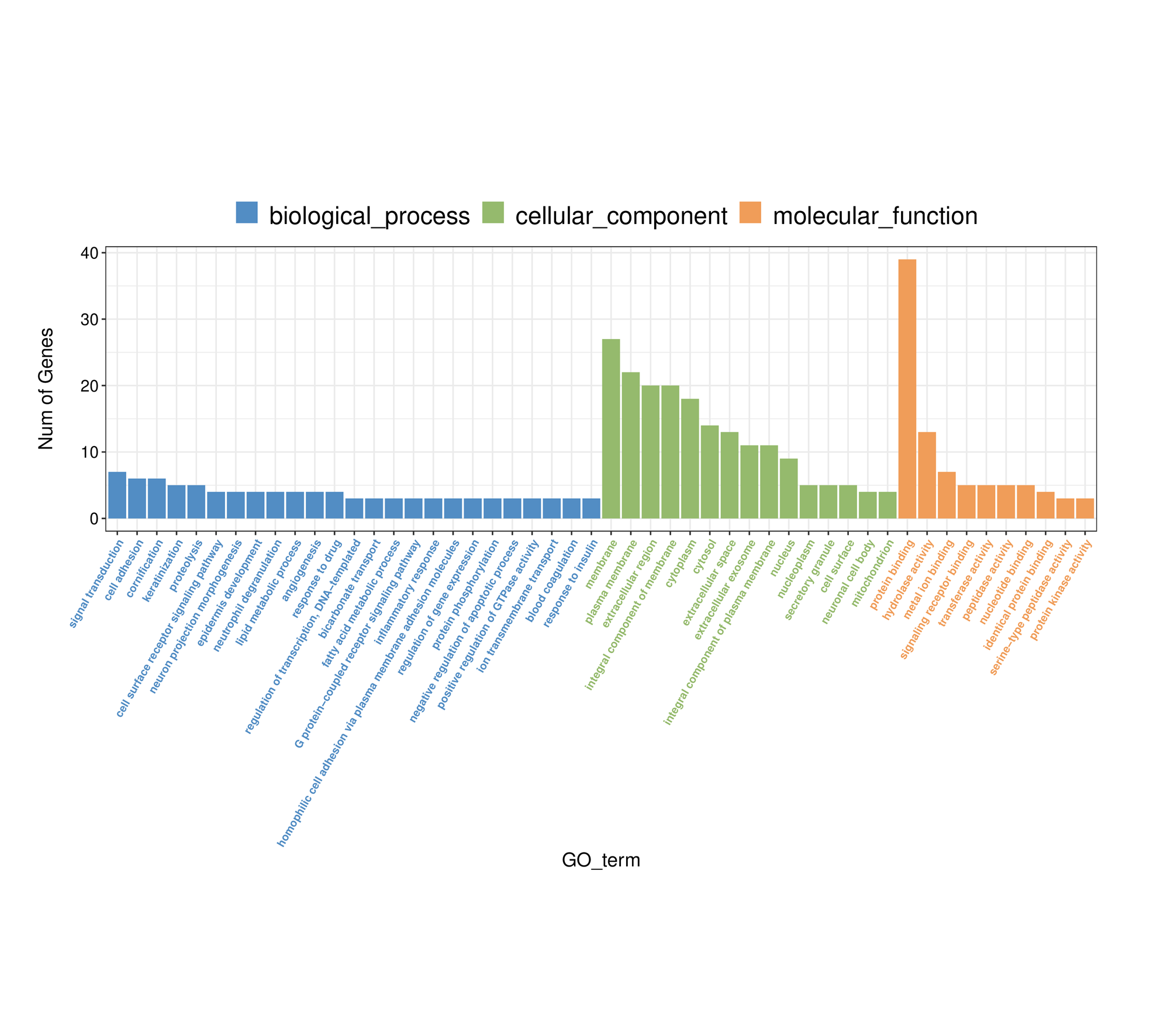

Supplement: Supplementary file 3 — SUPPORTING INFORMATION [file CTM2-12-e734-s002.tif]

**A**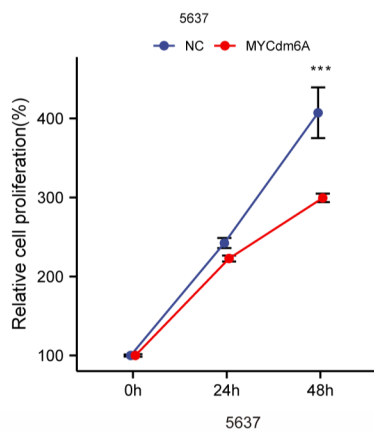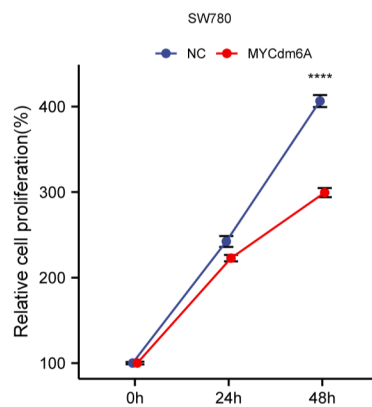**B**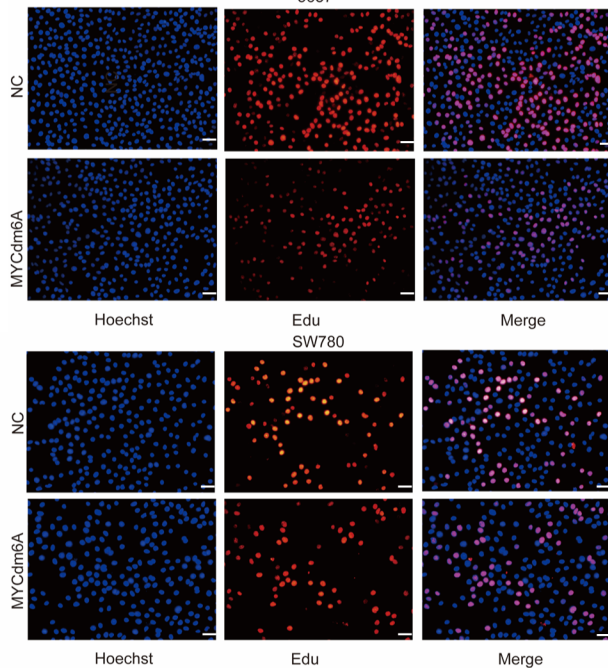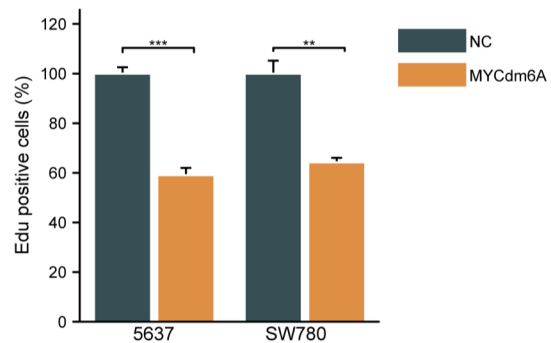

Supplement: Supplementary file 4 — SUPPORTING INFORMATION [file CTM2-12-e734-s001.pdf]
