# Supplementary material for: A synthetic targeted RNA demethylation system based on CRISPR‐Cas13b inhibits bladder cancer progression
Source: Clin Transl Med. 2022 Feb 27;12(2):e734. doi: 10.1002/ctm2.734 (PMC8882238; doi:10.1002/ctm2.734)
Supplement: Supplementary file 5 — SUPPORTING INFORMATION [file CTM2-12-e734-s004.docx]

**Materials and methods**

**Cell lines and cell culture**

Human BCa cell lines (5637 and SW780) were obtained from the Institute of Cell Biology, Chinese Academy of Sciences (Shanghai, China).Cell line 5637 was cultured in RPMI-1640 Medium (Gibco, USA).Dulbecco's modified Eagle's medium (Gibco, USA) was used to culture SW780 cells. The media were all supplemented with 10% fetal bovine serum (FBS, Gibco, USA). All cell lines were grown in a humidified incubator at 37 °C with 5% CO_2_.

**Construction of MYCdm6A and cell transfection**

Lentiviruses of MYCdm6A and the control lentivirus were chemically constructed by GeneChem Corporation (Shanghai, China).Transfection was carried out in accordance with the manufacturer's instructions.Puromycin (2 μg/ml, Sigma) was used for at least one week to identify stable clones. Cells were collected at the appropriate time periods for relevant assays.

**MeRIP-qPCR**

The relative abundance of the MYC mRNA in m6A antibody IP samples and input samples was determined using real-time qPCR.The leftover RNA was utilized for m6A-immunoprecipitation after keeping 500 ng RNA as an input sample. The m6A antibody (Synaptic Systems, Germany) was treated with 100 micrograms of RNA diluted in 500 μL IP buffer (150 mM NaCl, 10 mM Tris, pH 7.4, 0.1% NP-40). The ratio of its amount in IP to that in the input was used to calculate the relative m6A levels in MYC.

**RT-qPCR**

TRIzol reagent (Invitrogen, USA) was used to isolate total RNA from SW780 and 5637 cells according to the manufacturer's procedure.cDNAs of mRNA were synthesized using a Hifair™ II 1st Strand cDNA Synthesis SuperMix Kit (Yeasen, China)A Hieff UNICON® Universal Blue qPCR SYBR Green Master Mix (Yeasen, China) was used for real-time PCR.GAPDH was chosen as the endogenous control. Amplification was carried out using an ABI PRISM 7300 Fluorescent Quantitative PCR System using PCR mixtures made according to the manufacturer's instructions (Applied Biosystems, Foster City, CA, USA). The 2-△△CT technique was used to calculate expression fold changes. The primers for qPCR are mentioned in Table 1.

**Western Blot**

RIPA buffer mixed with a 1% protease inhibitor cocktail was used to lyse BCa cell lines (Millipore, USA).A BCA protein assay kit (Beyotime, China) was used to assess protein concentrations.SDS PAGE was used to separate cell lysates, which were then transferred to PVDF membranes (Millipore, USA).Membranes were blocked for 1 hour in TBST with 5% non-fat milk, then incubated overnight at 4° C with primary antibodies. Commercially available secondary antibodies conjugated to horseradish peroxidase were used and the interactions were visualized with chemiluminescence.

**Luciferase Reporter Assays**

According to the manufacturer's instructions, the MYC activity was measured using the MYC Reporter kit (BPS Biosciences, USA) and the Dual-Luciferase Reporter System (Promega, USA).The MYC dual-luciferase reporter vectors were transfected into cells seeded into 6-well plates (5 × 10^5^ cells per well). At 48 hours after transfection, luciferase activity was evaluated using the dual luciferase assay kit (Promega, USA). The activities of firefly luciferase were compared to those of Renilla luciferase.

**mRNA stability**

For the given time periods, cells were treated with 5 ug/ml actinomycin D (Act-D, Bioss, China) to block mRNA transcription. Following Act-D treatment, RNA was extracted from the cells using the TRIzol reagent (Invitrogen, USA). RNA levels were determined by qPCR and normalized with GAPDH.

**RNA immunoprecipitation assays**

The connection between MYC and YTHDF1 was determined using the Magna RIP RNA-Binding Protein Immunoprecipitation Kit (Millipore, USA) according to the manufacturer's instructions. The antibodies used in the test were as follows: anti-YTHDF1 (CST, USA), anti-MYC (CST, USA) and anti-lgG antibodies (provided in the RNA-Binding Protein Immunoprecipitation Kit).For cDNA synthesis, coprecipitated RNAs were utilized, and qRT-PCR was performed to assess the results.

**m6A-seq**

TRIzol (Thermo Fisher, USA) was used to isolate total RNA from cells. Following that, m6A sequencing was carried out (Lc-bio, China). ExomePeak, an R software program that detects m6A peaks in a bed or bam format, which can be displayed on the UCSC genome browser or IGV software, was given mapped reads from the immunoprecipitation (IP) and input libraries. HOMER was used to identify new and recognized motifs, and then Perl scripts were used to locate the pattern in relation to the peak summit. Then, by calculating fragments per kilobase of transcript per million, StringTie was utilized to calculate expression levels for all mRNAs from the input libraries.

**Cell apoptosis assays**

To identify cell apoptosis, we employed an ELISA test for caspase-3 and a flow cytometry assay. The human caspase-3 ELISA test kit (Cusabio, China) was used as directed to detect caspase-3 activity. Moreover, to examine these cells, we employed a flow cytometry kit (US Everbright Inc, China). The flow cytometry results were used to determine the percentage of dead cells (BD Biosciences, USA).

**Cell proliferation assays**

Cell proliferation was assessed using the Cell Counting Kit-8 (CCK-8) kit (Dojindo, Japan) and the ethynyl-2-deoxyuridine (EdU)-incorporation assay kit (Ribobio, China) as described in our prior research.

**Cell migration assays**

The wound-healing and Transwell migration tests were used to measure cell migration. Cells were sown at identical density in 6-well plates and cultivated to 80 percent confluency. Sterile pipette tips were used to create artificial gaps. Wounds were identified and photographed using a digital camera system.

**Animal models**

All animal experiments were approved by the the Ethics Committees of Shanghai Genechem Co.,Ltd, and all mice were handled humanely. At 4 weeks of age, twenty female BALB/c nude mice were kept in pathogen-free circumstances. To create the xenograft model, 10^4^ tumor cells were subcutaneously injected into the nude mice's right flank. We used a caliper to measure the tumor's length and breadth every three days. The mice were sacrificed at the end of the study, and tumor tissues were weighed.Hematoxylin and eosin (H&E) staining and Immunohistochemistry (IHC) detection were used to examine the tumor cells.

Supplementry Figure 1 Schematic diagram of MYCdm6A for targeted RNA demethylation.

(A) The overall survival in BLCA patients with high or low expression of MYC (TCGA)

(B) Map of lentivirus vectors.

(C) A schematic diagram of the whole mechanism.

(D) Confocal imaging with an antibody against FTO confirmed subcellular localisation of the fusion protein in 5637 cells. All scale bars, 50 µm.

Supplementary Figure 2 MYCdm6A decreases the binding between YTHDF2 and MYC mRNA in 5637 and SW780 cells. Data are shown as mean ± SEM. (**p*<0.05, ***p*< 0.01, ****p*< 0.001, *****p*< 0.0001).

Supplementary Figure 3 GO (Gene Ontology) enrichment study of m6A peaks with a significant change in the MYCdm6A group compared to the negative control group.

Supplementary Figure 4 MYCdm6A inhibited proliferation of BCa cell lines in vitro.

(A) CCK8 assay was used to assess the changes in proliferation in bladder cancer cell lines in vitro.

(B) Edu assay was used to assess the changes in proliferation in bladder cancer cell lines in vitro. All scale bars, 100 µm. Data are shown as mean ± SEM (**p*<0.05, ***p*< 0.01, ****p*< 0.001, *****p*< 0.001)

**Supplementary Table 1** Primers for PCR

| Gene | Forward Primers | Reverse Primers |
| --- | --- | --- |
| GAPDH | CGCTCTCTGCTCCTCCTGTTC | ATCCGTTGACTCCGACCTTCAC |
| MYC | GGCTCCTGGCAAAAGGTCA | CTGCGTAGTTGTGCTGATGT |
